# I’ve got a friend somewhere: control of social behavior across striatal subregions

**DOI:** 10.3389/fnbeh.2026.1763517

**Published:** 2026-02-17

**Authors:** Mona Xuan Li, Jinhee Baek, Michaela Y. Guo, Matthew B. Pomrenze, Allen P. F. Chen

**Affiliations:** 1 Department of Psychiatry and Behavioral Sciences, Stanford University, Stanford, CA, United States; 2 Feinberg School of Medicine, Northwestern University, Chicago, IL, United States

**Keywords:** autism spectrum disorder, dopamine, dorsal striatum, neuropsychiatric, nucleus accumben, serotonin, social behavior, tail of the striatum

## Abstract

Most animals and humans are inherently social, enabling group dynamics to promote survival. Despite their importance, how the brain calibrates appropriate social behaviors to maximize survival and benefits remains incompletely understood. Distributed networks of neural circuits mediate complex behavioral states, including social behaviors. The striatum has long-known to be a structure essential for motivation and goal-directed behavior. The striatum is massive: it extends far along the anterior-posterior axis and can be divided into ventral, dorsal, and posterior domains. While it is well-appreciated that these striatal domains control motivated behaviors through coordinated functions, such that ventral striatum (e.g., nucleus accumbens) governs motivation and rewards processing, dorsal striatum mediates motor planning and action selection, and the posterior striatum (i.e., tail of the striatum) integrates sensory inputs, much less is understood about how they modulate social interactions. This mini review discusses the current understanding of what aspects of social behavior are controlled by each striatal subregion. We focus on key studies that highlight prominent neuromodulators, such as dopamine, serotonin, and neuropeptides, and their roles in social behaviors. We propose a framework in which striatal subregions calibrate social interaction through coordinated activities that mediate distinct aspects of the social interaction, similar to general motivation. A deeper understanding of how distributed striatal circuits modulate social behavior will help inform the development of therapeutic approaches for social dysfunction in various psychiatric states.

## Introduction

Animals are inherently social, capable of forming relationships and influencing one another within their communities. Social behavior is foundational to not just survival but also has allowed human civilization to flourish. Different constructs, such as empathy and other prosocial behaviors, are becoming increasingly studied due to their relevance in neuropsychiatric disorders (Kennedy and Adolphs, 2012; Lamm et al., 2019). Although social behavior has been analyzed from many lenses, there remain many questions about its neural substrates, some of which may underlie psychiatric states.

There are several frameworks developed to understand the neural substrates of social control. The amygdala, known for its role in processing fear and emotions, has been shown to play a role in the automatic process, while the prefrontal cortex influences a more strategic response to environmental stimuli (Adolphs, 2009). In vertebrates, neuromodulators such as oxytocin and vasopressin are known to play a critical role in social behavior, though differences in sex and species must also be considered (Insel and Young, 2000). The mesolimbic rewards system, which includes structures such as the nucleus accumbens (NAc) in the striatum, is a key component of the neural circuitry that controls social behavior (O’Connell and Hofmann, 2011; Hart et al., 2014). Both human and primate studies support the idea that major dopaminergic pathways converge on the NAc and dorsal striatum (DS) in order to control general motivational behaviors (Haber and Knutson, 2010). Recently appreciated, the tail of the striatum (TS) has also recently been shown to have a distinct role in the striatum for processing sensory information, threat responses, and orienting toward salient stimuli (Valjent and Gangarossa, 2021; Green et al., 2024). Whether these major striatal subregions play synergistic or distinct roles in social behavior remains to be fully determined.

Impairment of social function is a characteristic of many psychiatric disorders, and many medications that improve sociability modulate serotonin (5-HT) or dopamine (DA) in striatal circuits (Lydiard and Bobes, 2000; Bedi et al., 2009; Chen et al., 2021). This therapeutic convergence highlights the striatum as a central neural hub for social motivation and suggests that targeting these pathways may be a promising strategy for treating disorders marked by social withdrawal or impaired social rewards. In this review, we discuss the known roles of the NAc, DS, and TS in shaping social behavior. We propose a framework in which the NAc, DS, and TS may contribute to different aspects of social behavior such as social rewards processing, action selection, and salience, all of which may integrate to govern social approach in specific contexts. Furthermore, dysfunction in these processes may underlie social deficits observed in autism, depression, and addiction (Walsh et al., 2021; Pomrenze et al., 2022b; Galiza Soares et al., 2024). Clarifying the contributions of these striatal subregions is essential for understanding the neural mechanisms that support social interaction and for identifying potential pharmacological targets to ameliorate social deficits in neuropsychiatric disorders.

## Nucleus accumbens

**FIGURE 1:**
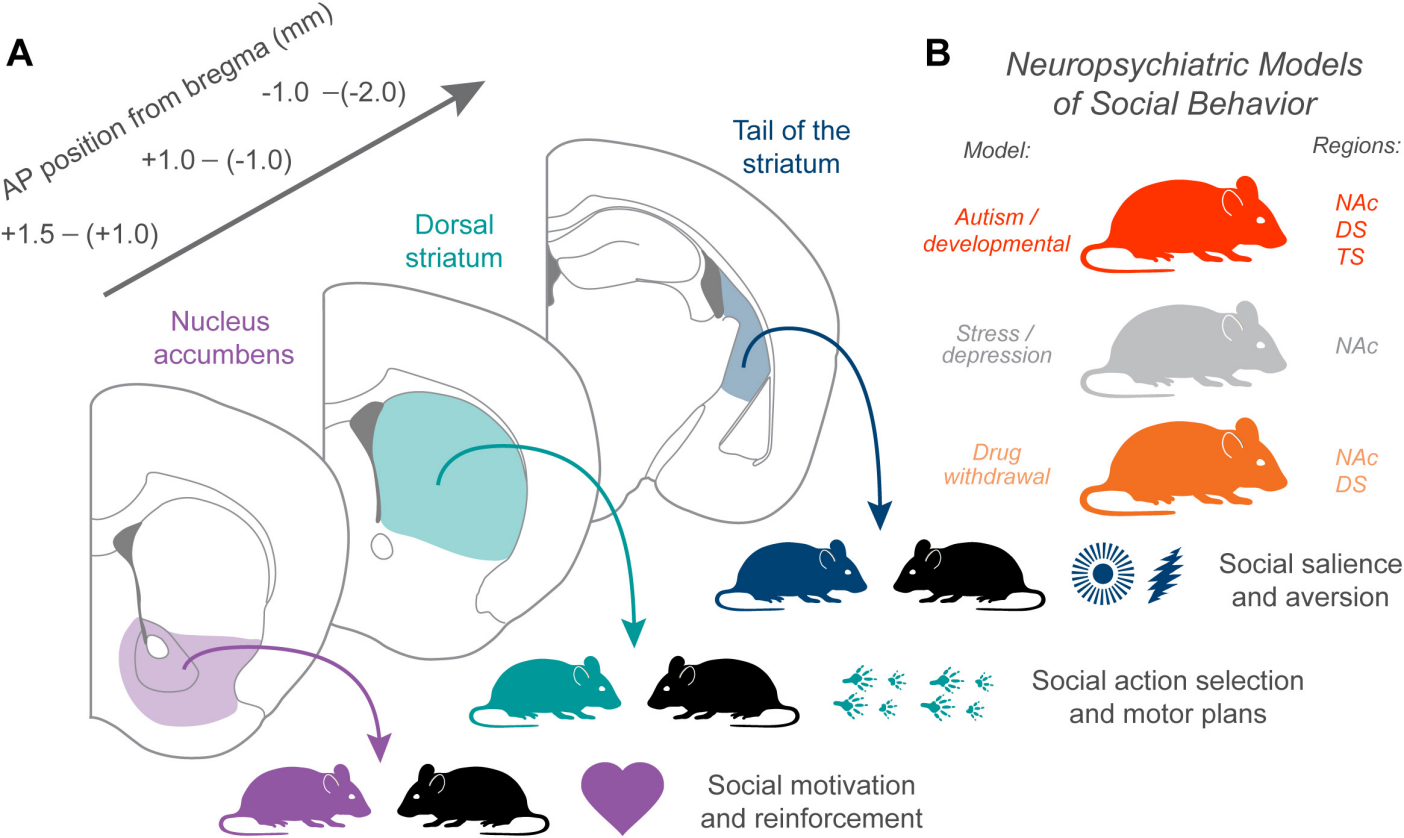
The contribution of different striatal subregions to social behavior and neuropsychiatric disease. **(A)** Coronal sections illustrate the anterior–posterior progression of major striatal territories: nucleus accumbens (NAc, purple), dorsal striatum (DS, teal), and tail of the striatum (TS, navy). Each region is linked to a distinct contribution to social function. NAc supports social motivation and reinforcement, dorsal striatum contributes to social action selection and motor policy execution, and TS may encode sensory identity and salience features that bias orienting toward or away from conspecifics. **(B)** Representative neuropsychiatric models associated with social dysfunction map onto these domains: autism and developmental etiologies often impair corticostriatal synaptic structure and social initiation, stress-related disorders alter rewards–valuation and social withdrawal, and drug withdrawal states influence social aversion and salience responses. Respective striatal regions implicated in each corresponding model are also highlighted here.

Amongst striatal subregions, the NAc remains the most well-studied in the context of social behaviors, owing to its central role in processing rewards, motivation, and affective states. Decades of work have shown that the NAc integrates diverse neuromodulatory inputs to shape how organisms evaluate and respond to social stimuli. This convergence of pathways positions the NAc as a critical node for translating the emotional and motivational value of social interactions into behavioral output. Studies across species demonstrate that disruptions in NAc signaling can affect social approach, affiliative behaviors, and social rewards learning, underscoring its importance in both typical social functioning and neuropsychiatric disorders marked by social deficits. The NAc is further divided into anatomically and functionally distinct subregions, the core and the shell. Their functional distinction in mediating incentive salience and emotional valence (Zahm, 2000; Floresco, 2015), respectively, suggests a similar potential dissociative role in social incentive processing and motivational value. However, their dissociative roles in complex social behavior have not been extensively addressed and remain to be further clarified. Combined, we here highlight work that suggests a collective role for the NAc in social rewards processing (Figure 1A).

In mice, the rewarding aspects of social interactions depend on the coordination of oxytocin (OT) and 5-HT in the NAc core (Dölen et al., 2013). Antagonizing oxytocin receptors (OTRs) prevents mice from showing preference for the social context in a social conditioned place preference assay, but did not affect cocaine preference. Additionally, OT induces long-term depression (LTD) of excitatory synapses on NAc MSNs, which requires the activation of presynaptic 5-HT1B receptors in the NAc. The dorsal raphe (DR) 5-HT neurons, the major source of serotonergic innervation to the NAc, are also well-known to modulate animal sociability in response to varying social experiences, such as social defeat (Challis et al., 2013, 2014). Accordingly, blocking the 5-HT1B receptors in the NAc core or genetically deleting OTRs from the DR abolishes social rewards, indicating that both neuromodulators are necessary for the reinforcing properties of social interaction. Consistent with this, in California mice, NAc core OTRs modulate the balance between social approach and social vigilance, primarily via Gq-coupled signaling cascades (Williams et al., 2020). This finding is supported and contextualized by a later study in which optogenetic stimulation of both serotonergic DR neurons and specifically NAc-projecting DR neurons were found to promote social approach (Walsh et al., 2018). Using the same manipulations, the authors were also able to rescue social behavior in an autism mouse model where chromosome 16p11.2 is deleted in mice, suggesting that targeted enhancement of NAc 5-HT signaling could be a therapeutic avenue for social dysfunction in autism spectrum disorders (ASDs) (Figure 1B). Similarly, it has been shown that 5-HT release in the NAc core is critical for empathy-like behavior in mice (Rein et al., 2024). This paper also showed that deletion of SHANK3, a postsynaptic density protein implicated in ASD, schizophrenia, and intellectual disability, led to a loss of empathetic behavior. Restoring 5-HT transmission in the Shank3−/− mice improved social and empathetic behaviors. As SHANK3 is critical for corticostriatal synapse structure and function, whether 5-HT plays a role in modulating corticostriatal inputs requires future investigation.

These principles of social modulation are further contextualized in the opioid system. Opioid withdrawal states are frequently accompanied by social withdrawal and related affective disturbances. Activating kappa opioid receptors (KORs) in the NAc medial shell inhibits 5-HT signaling, leading to maladaptive social withdrawal that may contribute to relapse in opioid-dependent individuals (Pomrenze et al., 2022a). Dynorphin-producing neurons in the DR release dynorphin onto KORs in the NAc, which suppresses 5-HT release during social encounters. Blocking dynorphin release from DR*Pdyn* neurons, or deleting KORs from 5-HT neurons, prevented the withdrawal-induced loss of sociability, whereas optogenetic activation of DR*Pdyn* neurons reproduces the KOR-dependent social deficits. This implicates NAc 5-HT and opioid signaling in drug withdrawal (Figure 1B). While 5-HT from the DR is critical for social interaction, it should be noted that the DR contains many other cell-types that release a host of other neuromodulators and neuropeptides, many of which contribute to addiction, but with unknown roles in social rewards or motivation (Pomrenze et al., 2021). For instance, some non-serotonergic DR neurons provide glutamatergic projections to midbrain dopamine neurons and drive rewards-related behavior (McDevitt et al., 2014). Similar to dynorphin, these other modulators may prove crucial for modulating social behavior in addiction or other disease states.

The NAc in other mammals, such as rats and prairie voles, also plays important roles in social behavior. In rats, balanced dopaminergic activity in the NAc is essential for social motivation (Manduca et al., 2016). Increases in extracellular DA through pharmacological manipulations suppress spontaneous social play, indicating that excessive DA disrupts this rewards-related behavior. Additionally, while antagonizing D2 receptors in the NAc reduces social play, agonizing D2Rs restores play levels after social isolation, which is consistent with the role of DA as a social rewards signal (Pomrenze et al., 2021). In another study done by Kopec et al. (2018), microglia and complement-mediated phagocytosis selectively eliminate dopamine D1 receptors (D1Rs) in the NAc of male rats, but not females during adolescence. This reveals that microglia-dependent synaptic remodeling of DA signaling underlies sex-differentiated maturation of rewards-related social behavior during a critical developmental window.

Prairie voles uniquely exhibit monogamous pair-bonding. The NAc plays a critical role in the formation of pair-bonding by regulating OT, DA, and their functional cooperativity. Notably, monogamous prairie voles exhibit significantly higher OTR density within the NAc compared to promiscuous meadow voles (Young et al., 2002; Loth et al., 2025). Without mating, partner preference in female voles can be induced by administering either OT or DA. In fact, access to both D2Rs and OT is required for this formation, as local D2R antagonism in the NAc shell blocks any partner preference induced by OT (Liu and Wang, 2003). Additionally, it was found that within the NAc, only DA in the rostral shell will promote pair bond formation, with D1Rs preventing the bond and D2Rs facilitating it (Aragona et al., 2006). The voles that exhibit pair bond maintenance and are aggressive to unfamiliar females also showed D1R upregulation, which indicates that DA in the NAc regulates motivated behavior in distinct ways. This is supported by a previous finding that injecting haloperidol, an antipsychotic that blocks DA, in the NAc shell of voles blocked partner preferences (Aragona et al., 2003). Also, injecting apomorphine, a DA agonist, in the NAc but not the caudate putamen induces preference for a social partner. Consistent with these findings in voles, optogenetic stimulation of OT inputs in the ventral tegmental area (VTA) increased the firing of NAc-projecting DA neurons and social approach in mice, indicating OT release in the VTA can act as a gate for social rewards (Hung et al., 2017). Together, these findings illustrate that dopaminergic and oxytocinergic signaling within the NAc forms a tightly coordinated system that underlies both the establishment and maintenance of pair bonds in prairie voles, mice, and likely other mammals (e.g., humans).

Canonical model of the NAc divides MSNs into D1 and D2 receptor–expressing principal subtypes, which are known to exert opposing influences on rewards processing and motivated behavior. This functional divergence is also evident in responses to chronic social defeat stress (CSDS) (Francis et al., 2015). Following CSDS, selective enhancement of D1 MSN activity promotes behavioral resilience, enabling animals to maintain normal social interaction despite stress exposure. Conversely, inhibition of D1 MSNs shifts animals toward a susceptible phenotype, characterized by social avoidance and anhedonia. In contrast, while manipulating D2 MSNs does not alter behavioral responses to standard CSDS, their repeated activation induces social avoidance following subthreshold social defeat stress, suggesting that D1 and D2 MSNs play distinct roles in shaping stress-related social outcomes.

Interestingly, D1 and D2 MSNs express numerous 5-HT receptors, although how different MSNs are modulated by 5-HT, or DA and 5-HT together, remains poorly understood. While both DA and 5-HT signaling are clearly important for social behavior, a recent study demonstrates that sucrose rewards conditioning is mediated by opposing rather than synergistic DA and 5-HT signals (Cardozo Pinto et al., 2025). This suggests context-dependent roles for these modulators and a distinct mechanism for social behavior. Anatomical analysis indicates that D1 MSNs express a larger degree of Gi-coupled 5-HT receptors while D2 MSNs express more Gs- and Gq-coupled 5-HT receptors (Pinto et al., 2025), suggesting antagonistic modulation by DA and 5-HT. Moreover, the enrichment of D2 MSNs in the NAc medial shell with 5-HT2c receptors suggests an important role for 5-HT via 5-HT2c in calibrating MSN activity, which has been proposed for the effects of MDMA rewards (Pomrenze et al., 2025), a drug with strong prosocial properties.

Collectively, this work underscores that finely tuned interactions among OT, 5-HT, DA, and related neuromodulatory pathways in the NAc and its upstream inputs are essential for maintaining adaptive social behavior. Overall, these findings suggest that the NAc and its neuromodulators orchestrate social rewards processing via mechanisms distinct from general motivation or rewards seeking, a property that could prove important for targeting social symptoms of psychiatric disease.

## Dorsal striatum

The dorsal striatum (DS), also known as the caudoputamen in mice and caudate/putamen structures in primates and humans, has long been known for its role in decision making, motor control, and learning (Balleine et al., 2007). As part of the basal ganglia, it is well-understood for its role in controlling movement (Albin et al., 1989), in learning and memory (Goodman and Packard, 2018), and decision making (Balleine et al., 2007). Increasing evidence now supports a complementary role for the DS in social behavior, mediated through corticostriatal plasticity, genetic influences, and neuromodulatory regulation. The DS can also be further divided into the dorsomedial and the dorsolateral striatum, which are functionally specialized for goal-directed actions and habitual behaviors (Yin and Knowlton, 2006), respectively. However, while these subregions likely contribute to distinct aspects of social interaction, the specific subregional contributions in social contexts are often not explicitly distinguished and require further investigation. Thus, we here review work that suggests a collective role for the DS in social action selection (Figure 1A).

Across rodents, primates, and humans, converging data suggest that the DS integrates social rewards signals and sensory inputs to support social decision-making and action selection (Balleine et al., 2007; Báez-Mendoza and Schultz, 2013; Báez-Mendoza et al., 2013). Consistent with this, using *in vivo* electrophysiology, it has been shown that striatal neurons encode social actions regardless of rewards (Báez-Mendoza et al., 2013). Importantly, this social action encoding eroded when taken out of the context of the social conspecific. Human neuroimaging work similarly shows dorsal striatal engagement during social observation, generosity, and social reinforcement (Harbaugh et al., 2007; Burke et al., 2010). In macaques performing tasks with both social and non-social rewards, caudate neurons, primarily in medial parts, are preferentially modulated by social rewards, whereas putamen neurons are more sensitive to primary fluid rewards (Klein and Platt, 2013), suggesting functional specialization within dorsal striatal territories for different motivational dimensions, similar to the NAc.

In contrast to primate literature, most rodent evidence for DS involvement in social behavior comes from neuropsychiatric disease models. Disruptions in corticostriatal synaptic scaffolding, especially those involving ASD-linked genes, such as SHANK3, produce parallel deficits in dorsal striatal physiology and social behavior. In the DS, Shank3−/− mice show altered MSN morphology, impaired corticostriatal synaptic transmission, robust reductions in social interaction, and repetitive grooming behavior (Peça et al., 2011). Consistent with this, distinct human-derived Shank3 mutations induce similar social deficits and dorsolateral striatal synaptic dysfunction (Zhou et al., 2016). Additional studies have shown that both homozygous and heterozygous Shank3 mutations induce sociability deficits, further validating the model (Jaramillo et al., 2017). In these studies, Shank mutations appear to reduce sociability in the contexts of 3-chamber sociability, novel juvenile interaction, abnormal ultrasonic vocalizations, and general dyadic interactions (less nose-to-nose contact and anogenital sniffing). Complementing this genetic work, a valproate-exposure model of ASD demonstrates altered dorsomedial striatal physiology, with D1-pathway MSNs showing elevated baseline activity yet reduced activation during social interaction (Di et al., 2022). Together, these findings indicate that disruptions in dorsal striatal postsynaptic scaffolding and corticostriatal integration can impair social behavior.

Neuromodulatory systems acting within the DS also influence social outcomes. Genetic pan-ablation of DA induces marked decreased motivation as well as social behavior (Hnasko et al., 2006). Rescue of DA specifically in the DS appears to be sufficient for rescue of maternal-pup interactions (Henschen et al., 2013). This may suggest that supraphysiological levels of DA lead to impaired social function. In addition to dopaminergic modulation, there is evidence that acetylcholine in the DS may play a role in social behavior. Cholinergic interneuron activity in the dorsomedial striatum has been shown to be important in mediating social hierarchy dynamics in the dominance tube test paradigm (Hsu et al., 2025). Consistent with this, ablation of cholinergic interneurons in the DS leads to aberrant, repetitive social exploration behavior (Martos et al., 2017). Together, these studies indicate that dopaminergic and cholinergic modulation of the DS play a role in normal social behavior and possibly social action selection.

## Tail of the striatum

The tail of the striatum (TS) is increasingly recognized as a specialized striatal domain for processing stimulus identity, value, and salience (Valjent and Gangarossa, 2021; Green et al., 2024). These functions are naturally relevant for recognizing conspecifics, tracking familiarity, and deciding whether to approach or avoid social cues, although only a few emerging studies have directly examined TS contributions to social behavior. Our current understanding points toward a model in which the TS may contribute to social behavior by representing the sensory identity and salience (Figure 1A).

Anatomically, TS receives widespread convergent input from sensory and value-coding regions. It is innervated by visual, auditory, somatosensory, and gustatory cortices, by thalamic sensory nuclei, by the basolateral amygdala, and by distinct sets of DA and 5-HT neurons (Hintiryan et al., 2016; Hunnicutt et al., 2016; Jiang and Kim, 2018). This organization positions TS as a site where detailed sensory identity information and motivational or value signals converge.

Recent work has defined a TS-specific DA subsystem. Menegas et al. (2018) showed that DA neurons projecting to the posterior TS form a distinct subclass whose activity reflects novelty and stimulus intensity rather than rewards. Perturbation of these neurons and associated downstream direct/indirect pathways reduces avoidance of threatening stimuli (Menegas et al., 2018; Tsutsui-Kimura et al., 2025). DA input to TS also contributes to auditory discrimination and fear learning, indicating a role in high-resolution stimulus identity coding and in shaping choices when sensory information is uncertain (Chen et al., 2022, 2023). Krüttner et al. (2022) demonstrate that in Shank3 mutant mice, absence of familiar contextual cues triggers enhanced DA transients in the TS, and that chemogenetic activation of prefrontal projections to TS restores normal engagement behavior. These findings suggest that TS may serve as a neural gate for social context familiarity and influences whether animals initiate or withdraw from an interaction. Furthermore, these findings also suggest a role for TS and TS DA in ASD deficits (Figure 1B), paralleling findings from the NAc (Walsh et al., 2018).

In primates, neurons in the caudate tail encode social identity directly. Kunimatsu et al. (2023) found cells that differentiate personally familiar from unfamiliar conspecific faces and that maintain long-term identity information. These findings suggest that the caudal striatum supports stable representations of social familiarity and identity. Green et al. (2024) have proposed a framework that unifies these observations in a general sense. They suggest that TS and its DA inputs participate in decisions to orient toward or away from salient stimuli. Behaviors such as object-directed saccades, tone-guided choices, and rapid defensive responses can all be viewed as expressions of a shared computation for which the TS may facilitate for social behavior.

## Conclusion and future outlooks

Social behavior arises from the integration of motivation, sensory identity, value, and action selection, processes distributed across many brain regions. In this review, we propose a functional framework in which the NAc, DS, and TS each contribute unique but complementary computations that together support adaptive social interaction. Furthermore, these mechanisms have been implicated in models of neuropsychiatric disease and social dysfunction (Figure 1).

The NAc remains the best-characterized striatal region in social neuroscience. Across rodents, voles, and humans, NAc integrates OT, 5-HT, and DA signals to generate social rewards, social approach, and pair bonding. As such, NAc provides a motivational substrate that energizes social engagement and links social cues to positive affective value. The DS, in contrast, contributes to the selection and execution of social actions. These findings position DS as the striatal locus where social action policies are selected, refined, and deployed, particularly in situations requiring rule learning or habits of social routines. The TS is emerging as a distinct sensory–value integration hub with clear implications for social behavior. Functionally, TS-specific DA signals convey novelty, intensity, familiarity, and threat-related salience rather than rewards valence. This tripartite framework, NAc for social rewards, DS for social action selection, and TS for social identity and salience, may help reconcile disparate findings across species and methodologies.

Despite progress, major gaps remain. NAc has been studied extensively with cell-type–specific tools, whereas DS and TS lack comparable causal and time-resolved interrogation during naturalistic social behavior. In particular, the TS has almost no direct causal evidence in social contexts beyond ASD-like models and primate identity coding. In addition, the contributions of neuromodulators beyond DA, such as OT and 5-HT in the DS and TS, remain largely unexplored. Future work combining ethologically rich paradigms with modern neural recording and manipulation tools will be essential to determine how these striatal territories interact to support social behavior. Clarifying these computations across NAc, DS, and TS may ultimately reveal how striatal microcircuits enable the flexible, context-dependent social behavior that is disrupted across many neuropsychiatric conditions.
